# Relationships between Ghrelin and Obestatin with MDA, Proinflammatory Cytokines, GSH/GSSG Ratio, Catalase Activity, and Semen Parameters in Infertile Patients with Leukocytospermia and Varicocele

**DOI:** 10.1155/2019/7261842

**Published:** 2019-10-21

**Authors:** Lucia Micheli, Giulia Collodel, Daniela Cerretani, Andrea Menchiari, Daria Noto, Cinzia Signorini, Elena Moretti

**Affiliations:** ^1^Department of Medical and Surgical Sciences and Neurosciences, University of Siena, Siena, Italy; ^2^Department of Molecular and Developmental Medicine, University of Siena, Siena, Italy; ^3^Department of Business and Law, University of Siena, Siena, Italy

## Abstract

Ghrelin and obestatin are involved in many biological functions including reproduction. Growing evidences suggest that both peptides could exert protective and antioxidant activities. In this study, the relationships between ghrelin/obestatin, interleukin-6 (IL-6), tumor necrosis factor-*α* (TNF-*α*), malondialdehyde (MDA), reduced glutathione (GSH), oxidized glutathione (GSSG), expressed as the GSH/GSSG ratio, catalase (CAT), and semen parameters in infertile patients with varicocele or leukocytospermia and controls were investigated. Fifty-six infertile patients (32 with leukocytospermia and 24 with varicocele) and 14 controls participated in this study. Semen analysis was performed following the WHO guidelines. Apoptotic and necrotic sperm were scored by annexin V/propidium iodide assay. Seminal plasma samples were used for the following determinations: ghrelin, obestatin, IL-6, and TNF-*α* were measured by an immunological method, GSH/GSSG by an enzymatic method, and CAT by spectrophotometric determination. With respect to controls, both the leukocytospermia and varicocele groups showed altered sperm parameters, significantly increased sperm apoptosis (*P* = 0.009 and *P* = 0.011, respectively), IL-6 (*P* = 0.0001 and *P* = 0.004, respectively), and TNF-*α* levels (*P* = 0.0001 and *P* = 0.002, respectively); both groups had significantly decreased levels of ghrelin (*P* = 0.0001), obestatin (*P* = 0.0001 and *P* = 0.006, respectively), and GSH/GSSG ratio (*P* = 0.003 and *P* = 0.0001, respectively). The MDA concentration was significantly increased in the leukocytospermia group *vs.* controls (*P* = 0.0001), in the varicocele group *vs.* controls (*P* = 0.011), and in the leukocytospermia group *vs.* the varicocele group (*P* = 0.008). CAT activity was augmented in both the leukocytospermia and varicocele groups (*P* = 0.0001)*vs.* controls. The results indicate that both ghrelin and obestatin may play a protective role in human semen and this effect is probably due to their antioxidant properties.

## 1. Introduction

Ghrelin and obestatin peptides are derived from a ghrelin-obestatin preproprotein that is cleaved to yield these two proteins. Although both hormones have the same precursor, their biological activities differ from each other. Various organs produce both hormones; however, most circulating ghrelin derives from A-like or Gr cells situated in the basal part of the glands' gastric oxyntic mucosa. It must be said, however, that ghrelin is more thoroughly investigated than obestatin [1]. Many biological functions such as regulation of food intake, gastrointestinal motility, sleep, cardiovascular functions, and cell proliferation are regulated by ghrelin [2]. It was initially proposed that obestatin exerts opposite effects to those of ghrelin by promoting satiety and thus decreasing the food intake [3]. However, growing evidences suggest that obestatin could be considered a sort of “protective” peptide as it may support cell survival and proliferation and could play a healing role in different organs [4]. Recently, much attention has been dedicated to the ability of ghrelin to reduce the inflammation processes by promoting a strong inhibition of inflammatory cytokine expression [5] and attenuating the oxidative stress in different organs [6–8]. Ghrelin exerts its antioxidant and anti-inflammatory effects by preventing the peroxidation and enhancing the activity of antioxidant enzymes [9]. Obestatin as well seems to exert antioxidant and antiapoptotic effects in several animal models used to study pathologies that have an inflammatory base [4, 10, 11].

Ghrelin, in particular, is found in both the male and female reproductive tracts together with its receptor called growth hormone secretagogue receptor-1a (GHSR-1a) [2, 12]. This suggests that ghrelin may have a role in the energy homeostasis control and reproduction [13]. In the male reproductive system, ghrelin is involved in key aspects of testis physiology, such as proliferation of Leydig cells, steroidogenesis, and regulation of hypothalamic-pituitary gonadal axis [14]. On the other hand, very little is known about obestatin, which was observed for the first time in rat Leydig cells [15]. Different animal models of testicular damage have been used to demonstrate the protective effect of ghrelin towards the oxidative stress [16–18]. The results suggested that the presence of such peptide in the reproductive sphere may play a new important role. At present, very few data on the putative activities of ghrelin and obestatin in the human semen is available. Years ago, some of our group demonstrated the presence of both ghrelin and obestatin in human semen samples and male genital tracts. The mean levels of obestatin in human semen were twenty times ca. higher than those measured in the serum of the homologous patients; the mean concentrations of semen ghrelin were twice as much those of serum ghrelin. In addition, we found that semen obestatin concentrations were positively correlated with sperm concentration and percentage of motile sperm, suggesting a possible protective role of this peptide [19]. A subsequent study reported the immunolocalization of both peptides in almost all organs of the human male reproductive system [20]. These observations prompted us to explore the potential protective effect of these peptide hormones in human semen. We determined the levels of ghrelin, obestatin, inflammatory cytokines such as interleukin-6 (IL-6) and tumor necrosis factor-*α* (TNF-*α*), malondialdehyde (MDA), a marker of oxidative stress, and antioxidant molecules such as reduced glutathione (GSH), oxidized glutathione (GSSG), expressed as GSH/GSSG ratio, and catalase (CAT). These variables were measured in semen of infertile patients grouped as follows: patients with leukocytospermia and patients with varicocele. The control group was composed of fertile males.

## 2. Materials and Methods

### 2.1. Patients

For this study, 56 infertile patients (aged 27-38) attending our laboratory for semen analysis were enrolled (from January through October 2018). All infertile patients did not obtain pregnancy after two years of unprotected sexual intercourses; the female factor was excluded.

The infertile patients were categorized into two groups: 32 infertile patients (aged 28-38) with leukocytospermia and 24 (aged 27-38) with varicocele. Leukocytospermia was detected during sperm analysis and defined as reported in the World Health Organization (WHO) guidelines [21]. The varicocele group was composed of patients who underwent both physical examination and scrotal colour Doppler ultrasonography analysis carried out in laboratories different from ours. For this study, we included patients with grade II and grade III varicocele.

All selected patients satisfied the following selection criteria: nonazoospermic men, 46, XY karyotype, and BMI < 25 kg/m^2^. Controls and patients had normal concentrations of follicle stimulating hormone (FSH), luteinizing hormone (LH), and testosterone (T). Hormone levels were assayed in serum by commercial kits (Beckman Coulter Access for FSH, LH, and testosterone). A normal range for FSH was 0.7-11.00 mU/mL (sensitivity 0.2 mUI/mL, intra- and interassay coefficient of variation <10%), for LH 0.8-8.0 mU/L (sensitivity 0.2 mUI/mL, intra- and interassay coefficient of variation <10%), and for T 2.7-10.9 mg/mL (sensitivity 0.1 ng/mL, intra- and interassay coefficient of variation <10%). The subjects considered in this study did not have genitourinary infections. Samples were seeded using a calibrated loop on agar plates, which were incubated overnight at 37°C in normal air with 5% CO_2_. The microorganisms were identified by gram stain, oxidase, catalase, and other biochemical tests using Bio-Mérieux products (Bio-Mérieux, Florence, Italy). Spermiocultures were considered positive when the number of colonies was ≥10^4^ CFU mL^−1^ in case of gram-positive cocci and ≥10^5^ CFU mL^−1^ in case of gram-negative rods.

The participants had no chronic diseases and did not receive radiotherapy, chemotherapy, and medication. None of the individuals took an oral antioxidant supplement for at least five months before the analysis. Subjects with a history of recreational drug use, alcohol consumption, and heavy smoking habit (>10 cigarette/day) were excluded in this study.

The control group was composed of 14 fertile men (aged 25-35) which fathered at least one child in the last 4 years. The fertile subjects showed a normal karyotype; they were not affected by infections and anatomical problems. Their semen parameters were higher than 25 percentiles as reported in the WHO guidelines [21].

All patients provided an informed written consent before the inclusion of this study. The informed consent describes the aims of the research, and it is approved by the Ethics Committee of Azienda Ospedaliera Universitaria Senese (CEAOUS).

### 2.2. Semen Analysis

Semen analysis was performed by a standard procedure according to the WHO guidelines [21]. Briefly, specimens were collected by masturbation after 3-5 days of sexual abstinence and analysed after liquefaction for 30 min at 37°C. Conventional semen parameters were determined: volume, pH, sperm concentration, and motility. The sperm morphology was assessed by the Papanicolaou (PAP) test modified for spermatozoa. Peroxidase stain was used to identify the leukocytes in semen samples. A leukocyte concentration ≥ 1 million cell/mL was considered abnormal [21]. In each sample, sperm apoptosis and necrosis were detected as subsequently described. Seminal samples were centrifuged (200g for 10 min); the supernatant composed of seminal plasma without spermatozoa was distributed in aliquots and stored at -80°C until different analyses were performed.

### 2.3. Detection of Sperm Apoptosis and Necrosis

The detection of sperm apoptosis and necrosis was performed by Vybrant apoptosis assay (Invitrogen Ltd., Paisley, United Kingdom) based on fluorescein isothiocyanate- (FITC-) annexin V (AnV, green fluorescence) and propidium iodide (PI, red fluorescence). AnV protein has affinity for phosphatidylserine that during the apoptotic process is translocated from the inner to the outer plasma membrane layer; PI stains necrotic cell with a broken membrane. The detailed procedure is described in Moretti et al. [22]; at least 300 sperm for each sample were analysed, and undamaged sperm (AnV negative, PI negative, not stained), apoptotic sperm (AnV positive, PI negative, green stained), and necrotic sperm (AnV negative, PI positive, red stained) were classified. Sperm with both green and red signals were scored as necrotic since their membrane was damaged.

### 2.4. Ghrelin, Obestatin IL-6, and TNF-*α* Determinations

Ghrelin, obestatin IL-6, and TNF-*α* were assayed in an aliquot of all 56 semen samples, after thawing. Ghrelin and obestatin concentrations were measured by radioimmunoassay using a Ghrelin (total) radioimmunoassay (RIA) kit (LINCO Research, St. Charles, Missouri, USA) and an obestatin (Human, Monkey) RIA kit (Phoenix Pharmaceuticals, Inc., Karlsruhe, Germany). The manufacturer's instructions were followed, and the results were expressed in ng/mL.

IL-6 and TNF-*α* concentrations were assessed by enzyme-linked immunosorbent assay (ELISA, Human IL-6 BMS213/2CE BMS213/2TENCE, and TNF-*α* BMS223/4CE BMS223/4TENCE; Bender MedSystems GmbH, Vienna, Austria), and the results were expressed in pg/mL. The calculation was done on the calibration curve as reported in the different kit manuals. For each sample, three specimens were determined.

### 2.5. Oxidized and Reduced Glutathione

After thawing, the seminal plasma (200 *μ*L) was added to an equal volume of 10% metaphosphoric acid. Samples were centrifuged at low speed (2,000g) for 10 min at 0°C. Total glutathione (GSH) and oxidized glutathione (GSSG) were quantified in a supernatant using a microassay procedure [23] using GSSG as the standard to define the calibration curve. Each sample was determined in triplicate, and the results were expressed in nmol of GSH or GSSG per mg of protein.

### 2.6. Catalase Activity

After thawing, the seminal plasma was centrifuged at 4,000g for 15 min at 4°C. To determine the catalase (CAT) activity, a microassay procedure was used [24].

This method, which employs 20 *μ*L, is based on the reaction of CAT with methanol in the presence of an optimal concentration of hydrogen peroxide. The formaldehyde production was measured spectrophotometrically at 540 nm with 4-amino-3-hydrazino-5-mercapto-1,2,4-triazole (Purpald, Sigma-Aldrich, Milan, Italy) as a chromogen. One unit of catalase activity was defined as the amount of enzyme that caused the formation of 1 nmol of formaldehyde per min at 25°C. Each sample was determined in triplicate, and the results were expressed as nmol/min/mg of protein.

### 2.7. Protein Assay

Protein concentrations were determined by the method of Lowry et al., and the calibration curves were prepared with dry bovine serum albumin [25].

### 2.8. Malondialdehyde Assessment

After thawing, the levels of lipid peroxidation in the seminal plasma were estimated using the evaluation of MDA levels. 500 *μ*L of samples were mixed with 500 *μ*L of a solution containing 0.04 M Tris (hydroxymethyl)methylamine (pH 7.4) and 0.01% butyl hydroxytoluene in acetonitrile were added (1 : 1, *v*/*v*) to prevent the artificial oxidation of polyunsaturated free fatty acids during the assay. The samples were centrifuged at 3,000g for 15 min. The supernatant was used for MDA high-performance liquid chromatography (HPLC) analysis with UV detection according to Shara et al. [26]. Each sample was determined in triplicate, and the results were expressed in nmol of MDA per mL of seminal plasma. The MDA samples were determined with respect to its own calibration curve.

### 2.9. Statistical Analysis

Statistical analysis was performed with the SPSS version 19 software package (SPSS Inc., Chicago, IL, USA). The Kolmogorov–Smirnov test was used to verify the normality of distribution of the variables investigated. Some variables (sperm progressive motility, obestatin, ghrelin, sperm morphology, necrosis, apoptosis, and GSH/GSSG ratio) were normally distributed, and the others (sperm concentration, IL-6, TNF-*α*, catalase, and MDA) were not normally distributed. These variables were transformed into a logarithmic form, obtaining values with a normal distribution. The assumption of homoscedasticity of the variance of groups was assessed using Levene's test. One-way analysis of variance (ANOVA) was used to identify the significant difference between the groups. Tukey's post hoc multiple comparison test was used under homoscedasticity instead, under heteroscedasticity, and the Games–Howell post hoc test was used. Since we included some subjects that smoke ≤ 10 cigarette/day, the data were adjusted for smoking. Spearman's rank correlation coefficient (rho) was used to assess significant correlation (positive or negative) between the studied variables. A *P* value less than 0.05 is considered significant.

## 3. Results

The 70 study selected participants were categorized as patients with leukocytospermia (#32), patients with varicocele (#24), and fertile controls (#14). The considered variables were compared between the three groups in order to explore if relationships between ghrelin, obestatin, cytokines, oxidative stress, and antioxidant ability of human semen could be related to infertility (Tables 1 and 2). The data were adjusted for “smoking” that did not result into a confounding variable.

Sperm concentration, motility, and morphology were significantly increased in the group of fertile men compared to those observed in both the leukocytospermia and varicocele groups (Table 1).

With respect to controls, the leucocytospermia and varicocele groups showed significantly increased sperm apoptosis (*P* = 0.009 and *P* = 0.011, respectively), IL-6 (*P* = 0.0001 and *P* = 0.004, respectively), and TNF-*α*(*P* = 0.0001 and *P* = 0.002, respectively) levels (Table 1).

Table 2 reports the comparisons of ghrelin and obestatin levels, MDA concentration, GSH/GSSG ratio, and CAT activity among the considered groups.

Both the leukocytospermia and varicocele groups showed significantly lower levels of ghrelin (*P* = 0.0001), obestatin (*P* = 0.0001 and *P* = 0.006, respectively), and GSH/GSSG ratio (*P* = 0.003 and *P* = 0.0001, respectively) compared to those measured in the control group.

The concentration of MDA was significantly increased in the leukocytospermia group versus controls (*P* = 0.0001) in the varicocele group versus controls (*P* = 0.011) and in the leukocytospermia group versus the varicocele group (*P* = 0.008). Finally, CAT activity was higher in both the leukocytospermia and varicocele groups (*P* = 0.0001) than that measured in the control group.

Table 3 reports the correlations between the measured variables calculated in the total group of the study participants (no. 70 subjects). As far as the sperm parameters are concerned, sperm concentration, motility, and normal morphology show significant positive correlations among them (*P* = 0.0001).

Sperm concentration positively correlates with ghrelin (*P* = 0.012) and GSH/GSSG (*P* = 0.0001) and negatively with necrosis (*P* = 0.012), TNF-*α* level (*P* = 0.004), IL-6 level (*P* = 0.019), MDA concentration (*P* = 0.050), and catalase activity (*P* = 0.0001).

Sperm motility shows positive correlations with obestatin (*P* = 0.001) and GSH/GSSG (*P* = 0.001) and negative correlations with apoptosis (*P* = 0.004), necrosis (*P* = 0.0001), TNF-*α* level (*P* = 0.001), IL-6 level (*P* = 0.003), MDA concentration (*P* = 0.015), and catalase activity (*P* = 0.0001).

Sperm normal morphology percentage positively correlates with ghrelin (*P* = 0.0001), obestatin (*P* = 0.018), and GSH/GSSG (*P* = 0.0001) and negatively with sperm apoptosis (*P* = 0.001), TNF-*α* level (*P* = 0.0001), IL-6 level (*P* = 0.0001), MDA concentration (*P* = 0.001), and catalase activity (*P* = 0.0001).

Sperm apoptosis exhibits positive correlations with TNF-*α*(*P* = 0.0001), IL-6 levels (*P* = 0.006), MDA concentration (*P* = 0.019), and catalase activity (*P* = 0.0001) and negative correlations with ghrelin (*P* = 0.002) and obestatin (*P* = 0.045).

Sperm necrosis positively correlates with IL-6 (*P* = 0.026) and negatively with GSH/GSSG (*P* = 0.018).

Ghrelin presents positive correlations with obestatin and negative correlations with TNF-*α* level, IL-6 level, MDA concentration (*P* = 0.0001), and catalase activity (*P* = 0.001).

Obestatin shows negative correlations with TNF-*α*(*P* = 0.001), IL-6 level (*P* = 0.002), MDA concentration (*P* = 0.0001), and catalase activity (*P* = 0.050).

TNF-*α* level positively correlates with IL-6 level (*P* = 0.0001), MDA concentration (*P* = 0.001), and catalase activity (*P* = 0.0001) and negatively with GSH/GSSG ratio (*P* = 0.050).

IL-6 level exhibits positive correlations with MDA concentration and catalase activity (*P* = 0.005 and *P* = 0.003, respectively) and negative correlations with GSH/GSSG ratio (*P* = 0.045).

Finally, catalase activity is positively correlated with MDA concentration (*P* = 0.004) and negatively with GSH/GSSG (*P* = 0.0001).

## 4. Discussion

The data presented in this paper are the first to show an interplay among ghrelin, obestatin, cytokines, MDA, GSH/GSSG ratio, and CAT activity in human semen. Some years ago, our group demonstrated that the human semen samples are particularly rich in ghrelin and obestatin with respect to serum samples [19] and that both hormones are produced by different organs and tissues of the male reproductive system [20]. Despite their massive presence, until now, the role of both hormones was unexplored in human semen. In the present study, ghrelin and obestatin positively correlated with semen parameters and each other and negatively with sperm apoptosis, IL-6 and TNF-*α* levels, MDA, and CAT. Moreover, infertile patients with leukocytospermia or varicocele showed significantly reduced seminal concentrations of obestatin and ghrelin concomitant with increased levels of CAT, MDA, IL-6, and TNF-*α* with respect to those observed in the control group. Therefore, we may hypothesize a relationship between an oxidizing environment and the levels of ghrelin and obestatin. Leukocytospermia and varicocele are both conditions associated with oxidative stress, as a consequence of inflammatory situations [27–30], and characterized by increased levels of cytokines [29, 31–33]. TNF-*α* seems to play a role in the pathogenesis of varicocele probably inducing sperm apoptosis [33], which, also in this study, was significantly increased in both groups of infertile patients with respect to controls. In both leukocytospermia [34] and varicocele [27, 30], the involvement of oxidative stress by the increased production of ROS was demonstrated. In this research, we confirmed that leukocytospermia and varicocele are characterized by an impairment of redox status because the GSH/GSSG ratio [35] was decreased and negatively correlated with cytokines, MDA, and CAT. GSH scavenges the excess of ROS and is oxidized to the GSSG form; in addition, GSH reacts with cytotoxic aldehydes, products of lipid peroxidation (LPO), and can protect the sperm plasma membrane [36]. LPO was detected by MDA evaluation and antioxidant buffering capacity by enzymatic CAT activity involved in the decomposition of H_2_O_2_ into water and oxygen, thus preventing LPO and improving sperm motility [37]. The rise of CAT activity in semen of infertile patients with varicocele and leukocytospermia could be explained as a possible “chronic oxidative stress” [38]. Despite the increased CAT activity in the semen of both groups of infertile patients, MDA levels were still elevated, suggesting that the CAT activity was not able to counteract the peroxide excess. Even though other authors reported a decreased CAT activity in infertile patients with varicocele [39, 40], the results of this study are similar to those obtained in a previous research of ours [35].

The ability of ghrelin to attenuate inflammation and its potent inhibitory effect on the expression of proinflammatory cytokines was recently reported [5] in districts other than the male reproductive system. Despite a quite rich literature on the multiple roles of ghrelin, very few data is available on the function played by obestatin either in animal models or in humans. It has been established that obestatin plays a role in increasing cell survival and proliferation and counteracting apoptotic process and inflammation [41]. Regarding the reproductive system, a protective activity of ghrelin was observed in different researches, performed in animal models, demonstrating that this peptide can decrease the testicular damages [17, 42–44]. Recent studies deal with the defensive role of ghrelin that can exert beneficial activity on redox imbalance for its anti-inflammatory and antioxidant properties also in the male reproductive system [18, 45–47]. In a rat model of varicocele, Asadi et al. [45] demonstrated that ghrelin can decrease the oxidative stress (evaluated by means of MDA) caused by varicocele and enhance the activity of antioxidant enzymes. In addition, ghrelin attenuates the negative effects of cyclophosphamide, a chemotherapeutic and immune-suppressor drug, upon sperm parameters by reducing oxidative stress and LPO of sperm membranes and by enhancing the antioxidant activity [18]. These recent reports are extremely in accordance with our observations made in human semen.

Therefore, it is possible to hypothesize a protective antioxidant role of ghrelin and obestatin also in human semen, even though in this study we did not explore the mechanism of action. In this research, another interesting observation regards the relationship between ghrelin/obestatin and sperm apoptosis. In human semen, decreased levels of ghrelin and obestatin are concomitant with an increase of sperm apoptosis, indicating a possible antiapoptotic activity of both hormones. The antiapoptotic effect of ghrelin is reported in the literature, for example, in neuronal cells [48] and in the male reproductive system also. Ghrelin showed notable anti-inflammatory and antiapoptotic effects in a rat testicular ischemia reperfusion model by decreasing IL-6 and TNF-*α* levels [47]; Kheradmand et al. [49] used a rat model of scrotal hyperthermia and demonstrated that ghrelin downregulated Bax expression, a protein involved in apoptotic process, and concomitantly upregulated PCNA, which is involved in cell proliferation.

## 5. Conclusions

We demonstrated that ghrelin and obestatin concentrations are reduced in patients with leukocytospermia and varicocele and both hormones are correlated with redox imbalance. Obviously, we do not expect that the results of the present study are simply translated to the clinical field. These data can add insights to understand the complexity of the different mechanisms involved in spermatogenesis and functioning of the male reproductive system.

## Figures and Tables

**Table 1:** Means and standard errors of semen parameters, apoptosis, necrosis, IL-6, and TNF-*α* assayed in semen samples of 70 men divided into 3 groups according to their clinical diagnoses and controls. Statistical methods are indicated, and the exact values of *P* are reported.

	Diagnosis			Statistics	
Variables	Leukocytospermia (L no. 32)	Varicocele (V no. 24)	Fertile men (F no. 14)	HDS Tukey's post hoc test (*P* value)	Games-Howell post hoc test (*P* value)
Sperm (mL × 10^6^)	57.12 ± 5.55	70.89 ± 5.76	171.68 ± 23.36	—	F *vs.* L (*P* = 0.001); F *vs.* V (*P* = 0.002)

Progressive motility (%)	30.47 ± 1.79	29.21 ± 2.39	51.29 ± 1.18	F *vs.* L (*P* = 0.0001); F *vs.* V (*P* = 0.0001)	—

Normal morphology (%)	8.69 ± 0.27	8.88 ± 0.37	16.79 ± 0.54	F *vs.* L (*P* = 0.0001); F *vs.* V (*P* = 0.001)	—

Apoptosis (%)	13.84 ± 1.05	14.00 ± 1.53	7.71 ± 0.13	F *vs.* L (*P* = 0.009); F *vs.* V (*P* = 0.011)	—

Necrosis (%)	17.41 ± 1.12	19.00 ± 2.91	13.64 ± 2.14	—	—

IL-6 (pg/mL)	14.75 ± 2.63	15.41 ± 3.48	1.86 ± 0.49	—	F *vs.* L (*P* = 0.0001); F *vs.* V (*P* = 0.004)

TNF-*α* (pg/mL)	36.21 ± 3.38	46.59 ± 8.74	11.82 ± 1.57	—	F *vs.* L (*P* = 0.0001); F *vs.* V (*P* = 0.002)

**Table 2:** Means and standard errors of ghrelin, obestatin, MDA, GSH/GSSG ratio, and catalase assayed in semen samples of 70 men divided into 3 groups according to their clinical diagnoses and controls. Statistical methods are indicated, and the exact values of *P* are reported.

Variables	Diagnosis			Statistics	
	Leukocytospermia (L no. 32)	Varicocele (V no. 24)	Fertile men (F no. 14)	HDS Tukey's post hoc test (*P* value)	Games-Howell post hoc test (*P*-value)
Ghrelin (ng/mL)	0.92 ± 0.03	0.916 ± 0.043	1.12 ± 0.047	F *vs.* L (*P* = 0.0001); F *vs.* V (*P* = 0.0001)	—

Obestatin (ng/mL)	4.00 ± 0.28	4.40 ± 0.33	6.01 ± 0.29	F *vs.* L (*P* = 0.0001); F *vs.* V (*P* = 0.006)	—

MDA (nmol/mL)	8.59 ± 1.27	3.66 ± 0.85	0.55 ± 0.05	—	F *vs.* L (*P* = 0.0001); F *vs.* V (*P* = 0.011); L *vs.* V (*P* = 0.008)

GSH/GSSG (nmol/mg of proteins)	10.38 ± 0.74	9.02 ± 0.73	14.58 ± 0.85	F *vs.* L (*P* = 0.003); F *vs.* V (*P* = 0.0001)	—

CAT (U/mg of proteins)	9.72 ± 1.26	10.21 ± 0.76	2.96 ± 0.46	—	F *vs.* L (*P* = 0.0001); F *vs.* V (*P* = 0.0001)

**Table 3:** Correlations (Spearman's coefficient (rho)) between all considered variables in 70 individuals. The exact values of *P* are reported in parentheses.

	Sperm (mL × 10^6^)	Motility (%)	Normal morphology (%)	A (%)	N (%)	Ghrelin	Obestatin	TNF-*α*	IL-6	MDA	CAT	GSH/GSSG
Sperm/mL × 10^6^	1											
Motility (%)	0.576 (.0001)	1										
Normal morphology (%)	0.509 (.0001)	0.475 (.0001)	1									
A (%)	-0.136 (ns)	-3.41 (.004)	-0.377 (.001)	1								
N (%)	-0.298 (.012)	-0.530 (.0001)	-0.166 (ns)	0.007 (ns)	1							
Ghrelin	0.299 (.012)	0.172 (ns)	0.421 (.0001)	-0.359 (.002)	0.076 (ns)	1						
Obestatin	0.122 (ns)	0.417 (.001)	0.282 (.018)	-0.241 (.045)	0.068 (ns)	0.639 (.0001)	1					
TNF-*α*	-0.338 (.004)	-0.389 (.001)	-0.553 (.0001)	0.751 (.0001)	-0.001 (ns)	-0.454 (.0001)	-0.396 (.001)	1				
IL-6	-0.324 (.019)	-0.399 (.003)	-0.577 (.0001)	0.370 (.007)	0.309 (.026)	-0.496 (.0001)	-0.422 (.002)	0.546 (.0001)	1			
MDA	-0.257 (.050)	-0.344 (.015)	-0.468 (.001)	0.334 (.019)	0.0168 (ns)	-0.502 (.0001)	-0.506 (.0001)	0.452 (.001)	0.443 (.005)	1		
CAT	-0.732 (.0001)	-0.562 (.0001)	-0.690 (.0001)	0.548 (.0001)	0.194 (ns)	-0.525 (.001)	-0.304 (.050)	0.626 (.0001)	0.540 (.003)	0.491 (.004)	1	
GSH/GSSG	0.515 (.0001)	0.420 (.001)	0.476 (.0001)	-0.096 (ns)	-0.309 (.018)	0.149 (ns)	0.105 (ns)	-0.255 (.050)	-0.315 (.045)	-0.185 (ns)	-0.551 (.0001)	1

Legend: Sperm/mL × 10^6^: number of sperm/ml; Motility (%): percentage of progressive sperm motility; Normal morphology (%): percentage of normal sperm morphology assessed with Papanicolaou staining; A (%): percentage of sperm apoptosis assessed with AnV/PI assay; N (%): percentage of sperm necrosis assessed with AnV/PI assays; Ghrelin (ng/mL); Obestatin (ng/mL); TNF-*α* (pg/mL): tumor necrosis factor-*α* (pg/mL); IL-6: interleukin 6 (pg/mL); MDA (nmol/mL): malondialdehyde (nmol/mL); GSH (nmol/mg): glutathione (nmol/mg of protein); GSSG (nmol/mg): glutathione oxidized form (nmol/mg of protein); CAT (U/mg): catalase activity (U/mg of protein).

## Data Availability

The data used to support the findings of this study are included within the article.

## Abbreviations

IL-6: Interleukin-6
TNF-*α*: Tumor necrosis factor-*α*
MDA: Malondialdehyde
GSH: Reduced glutathione
GSSG: Oxidized glutathione
CAT: Catalase
WHO: World Health Organization
FSH: Follicle stimulating hormone
LH: Luteinizing hormone
T: Testosterone
PAP: Papanicolaou
FITC: Fluorescein isothiocyanate
AnV: Annexin V
PI: Propidium iodide
ELISA: Enzyme-linked immunosorbent assay
RIA: Radioimmunoassay
HPLC: High-performance liquid chromatography
ROS: Reactive oxygen species
LPO: Lipid peroxidation.
